# Characteristics of the Anterior Segment Biometry and Corneal Endothelium in Eyes with Pseudoexfoliation Syndrome and Senile Cataract

**DOI:** 10.4274/tjo.48264

**Published:** 2015-10-05

**Authors:** Banu Bozkurt, Hüseyin Güzel, Ümit Kamış, Şansal Gedik, Süleyman Okudan

**Affiliations:** 1 Selçuk University Faculty of Medicine, Department of Ophthalmology, Konya, Turkey; 2 Bitlis State Hospital, Clinic of Ophthalmology, Bitlis, Turkey

**Keywords:** pseudoexfoliation glaucoma, senile cataract, confocal microscopy, cornea endothelial cell density, optical low-coherence reflectometry

## Abstract

**Objectives:**

To evaluate the anterior segment biometric features and corneal endothelial changes in eyes with pseudoexfoliation (PEX) syndrome and senile cataract.

**Materials and Methods:**

The central corneal thickness, anterior chamber depth (ACD), pupil diameter, lens thickness, endothelial cell density (ECD), and percentages of polymegathism and pleomorphism of 52 subjects with PEX and cataract were compared with 51 age- and gender-matched control subjects with cataract using optical low-coherence reflectometry (OLCR, Lenstar LS 900; Haag Streit AG, Switzerland) and in-vivo confocal microscopy (Confo Scan 4, Nidek Co. Ltd, Osaka, Japan). Nineteen subjects with PEX syndrome had glaucoma and were using anti-glaucoma medications. Only one eye of the subjects was used in statistical analysis and a p value less than 0.05 was considered statistically significant.

**Results:**

None of the OLCR parameters reached statistically significant differences among the 3 groups (ANOVA p>0.05). The percentage of eyes with ACD <2.5 mm was 13.7% in the control group, 24.2% in PEX eyes without glaucoma and 21.1% in PEX eyes with glaucoma, with no statistically significant differences (p=0.45). There was a significant difference in mean ECD among the 3 groups (ANOVA p=0.02), whereas no differences could be found in respect to polymegathism and pleomorphism (p>0.05). Mean ECD was significantly lower in the PEX glaucoma group (2,199.5±176.8 cells/mm2) than the control group (2,363±229.3 cells/mm2) (p=0.02), whereas no difference was found in mean ECD of PEX eyes without glaucoma and the control group (p=0.42). ECD was less than 2,000 cells/mm2 in 15.8% of PEX subjects with glaucoma, 9.8% of control subjects and 6.1% of PEX eyes without glaucoma, with no statistically significant difference (p=0.52).

**Conclusion:**

As eyes with both PEX glaucoma and cataract seem to be associated with decreased endothelial cell number, specular or confocal microscopy screening should be done for the patients scheduled for intraocular surgery.

## INTRODUCTION

Pseudoexfoliation (PEX) syndrome is an age-related disorder of the extracellular matrix characterized by the production and progressive accumulation of abnormal extracellular fibrillar material in the inner wall tissues of the anterior segment of the eye including the lens capsule, iris, non-pigmented ciliary epithelium, trabecular meshwork and corneal endothelial cells.^1^ This accumulation predisposes the eye to a broad spectrum of intraocular complications including cataract with zonular instability, lens decentration, secondary open-angle glaucoma, angle-closure glaucoma, melanin dispersion and iridopathy with a smaller pupilla.^1,2,3,4,5^ Zonular instability may lead to intraoperative complications during cataract surgery, most notably zonular dialysis and vitreous loss.^3,4,5,6,7,8^ Previous studies showed that zonular weakness with subsequent phacodonesis in PEX syndrome could lead to anterior lens movement, increased lens thickness (LT) and shallow anterior chamber.^6,7,8^ In eyes with PEX syndrome, a small anterior chamber depth (ACD) less than 2.5 mm was found to be associated with a risk of 13.4% for intraoperative complications compared with an incidence of 2.8% for an ACD of 2.5 mm or more.^6^ Anterior chamber was found to be significantly shallower in PEX eyes with intraoperative complications (zonular dialysis, and/or vitreous loss) compared to PEX eyes with no complications (p<0.05).^9^ It is therefore very important for the cataract surgeon to predict which eyes are at risk for developing intraocular complications. It is possible to detect these ocular biometric changes earlier using imaging technologies with objective and quantitative measures, such as A scan ultrasound, optical low-coherence reflectometry (OLCR) and optical coherence tomography (OCT). Lenstar LS 900® (Haag-Streit, Switzerland), the newest generation of OLCR, provides true A-scan measurements of optical ocular components in a single shot without contacting the eye.^10,11,12^ PEX syndrome has been found to be associated with cornea endotheliopathy in some histopathological studies, which has been suggested to be the cause of the so-called atypical non-guttata Fuchs endothelial dystrophy.^13,14^ Decreased endothelial cell density (ECD) has also been confirmed in eyes with PEX syndrome using specular and confocal microscopy.^15,16,17,18,19^ On the other hand, some investigators did not find evidence of this association.^20,21,22^

In this study, our aim was to evaluate anterior segment biometry using OLCR and corneal endothelial changes with confocal microscopy in PEX syndrome/glaucoma eyes before cataract surgery and compare these findings with cataractous eyes without PEX syndrome.

## MATERIALS AND METHODS

The study was conducted in the Department of Ophthalmology, Selçuk University Faculty of Medicine in accordance with the tenets of the Declaration of Helsinki, and the study protocol was approved by the local ethics committee (23/07/2013, 2013/235). All patients underwent a detailed ophthalmic assessment, including best corrected visual acuity measurement, intraocular pressure (IOP) measurement by Goldmann applanation tonometry, slit-lamp biomicroscopy, gonioscopy, and fundus examination. PEX was defined as the presence of pseudoexfoliative material in either eye at the pupil margin, on the lens capsule, or both. After a detailed high-magnification slit-lamp assessment of the pupil margin, the pupil was dilated and the anterior lens surface was examined for pseudoexfoliative material on the lens capsule and also for early signs of PEX syndrome, including pre-granular radial lines and granular deposits. The diagnosis of PEX glaucoma was based on the findings of an elevated IOP (>21 mmHg), typical glaucomatous cupping of the optic nerve head, and visual field defects.

Fifty-two subjects with PEX were compared with 51 age- and gender-matched control subjects. Nineteen subjects with PEX syndrome had glaucoma and were using anti-glaucoma medications. Subjects with previous intraocular surgery, contact lens use, corneal trauma or other corneal pathologies which might affect the corneal endothelium, including Fuchs’ endothelial dystrophy, were excluded from the study. None of the glaucoma subjects were using topical miotics.

The Lenstar LS 900 performs the biometry of the whole eye at approximately 20 seconds per measurement using a 820 nm superluminescent diode. Besides keratometry and intraocular lens power, this OLCR instrument also measures central corneal thickness (CCT), ACD, pupil diameter (PD), LT, axial length and retinal thickness. In the examination with Lenstar LS 900®, the subjects were asked to place their chin on the chinrest, and lean their forehead against the headrest of the device. They were instructed to blink immediately before the measurements were taken, to obtain an optically smooth tear film over the cornea. The subjects were then asked to gaze at the round circle in front of them and not to blink during the measurement. The device detected blinking or loss of fixation automatically, and the measurements were repeated in this case. At least three measurements were taken and the best one was chosen for statistical analysis. The CCT, ACD, PD and LT, which were calculated automatically by the internal software of the device, were recorded and interpreted.

Confocal microscopy (ConfoScan 4.0, Nidek Co Ltd, Osaka, Japan) was performed by the same experienced technician and under the same conditions. The patients were positioned appropriately and asked to focus on an internal fixation light. The objective (20x) was then brought closer to the eye that was being examined, without touching the cornea. After the levels of the corneal cells had been recognized, a scanner performed the examinations automatically. During the scanning process, the images can be viewed at the scanner monitor. Images of different levels of the cornea were recorded on the computer. ConfoScan 4 has an endothelial analysis program that identifies polygonal representations of the cells by using proprietary image-processing routines and contains an internal calibration for magnification. From these images, the ratios between the ECD, pleomorphism (variation in cell shape from 6-sided endothelial cells, loss of hexagonality), and polymegathism (coefficient of variation of cell area) were calculated automatically. The mean of three sections was used for statistical analysis. The endothelial cell count was calculated automatically.

Statistical analysis was performed using SPSS for Windows version 18.0 (SPSS Inc., Chicago, IL, USA) and only one eye of the subjects was used in the statistical analysis. If both eyes were eligible for the study, one eye was chosen randomly for the study. If only one eye was eligible for study, that eye was used in the statistical analysis. Normality for continued variables was determined by the Shapiro-Wilk test. The parameters showing normal distribution were compared between PEX eyes without glaucoma, PEX eyes with glaucoma and control eyes using ANOVA. In case of significance, the parameters were compared again between the subgroups using the Tukey HSD post hoc test. The descriptive statistics were shown as mean ± standard deviation. The gender and the percentage of eyes with ACD <2.5 mm and ECD <2,000 cells/mm2 were compared among the three groups using the chi-square test. Level of significance was accepted as α=0.05.

## RESULTS

There were 16 men (48%) and 17 women (52%) among the PEX subjects without glaucoma, 12 men (63%) and 7 women (37%) among the PEX subjects with glaucoma and 28 men (55%) and 23 women (45%) in the control group, with no statistically significant difference (p=0.59). The mean ages of the PEX syndrome without glaucoma, PEX glaucoma and control groups were 69.15±7, 69.37±9.5 and 67±5.7 years, respectively, with no statistically significant difference (p=0.26). In the glaucoma group, 4 subjects were using one topical anti-glaucoma medication, 7 subjects were using 2, 6 subjects were using 3, and 2 subjects were using 4.

None of the OLCR parameters reached statistically significant differences among the 3 groups (ANOVA p>0.05). The percentage of eyes with ACD <2.5 mm was 13.7% in the control group, 24.2% in PEX eyes without glaucoma and 21.1% in PEX eyes with glaucoma, with no statistical significant differences (p=0.45).

There was a significant difference in mean ECD among the 3 groups (ANOVA p=0.02), whereas no differences could be found with respect to polymegathism and pleomorphism (p>0.05) (Table 1). Mean ECD was significantly lower in PEX glaucoma eyes (2,199.5±176.8 cells/mm2) than in the control eyes (2,363±229.3 cells) (p=0.02), whereas no difference was found in mean ECD between PEX eyes without glaucoma and the control group (p=0.42) (Table 1). ECD was less than 2,000 cells/mm2 in 15.8% of PEX subjects with glaucoma, compared to 9.8% in control subjects and 6.1% in PEX eyes without glaucoma, with no statistical significant difference (p=0.52).

## DISCUSSION

The results of studies in the literature concerning the characteristics of anterior segment biometry of eyes with PEX syndrome are controversial. Some studies found significantly shallower anterior chambers in eyes with PEX compared to age- and gender-matched patients without PEX syndrome and primary open-angle glaucoma.^7,8,23,24^ Bosnar et al.^7^ compared the optical ocular features of 47 eyes with cataract complicated by PEX syndrome with 177 eyes with uncomplicated cataract using the Lenstar LS 900® OLCR instrument. ACD was lower (t=-2.24, p<0.05) and LT (t=3.01, p<0.001) was higher in the PEX group (2.43±0.38, 4.70±0.39, respectively) compared to the control group (2.59±0.45, 4.43±0.57, respectively). In a study by Damji et al.,^8^ anterior chamber was found to be progressively shallower in PEX eyes having open or occludable angle in comparison with eyes primary open-angle glaucoma. A recent study by AS-OCT showed that PEX eyes had significantly smaller anterior chamber angle, shallower ACD, greater iris convexity and thinner irises than their fellow eyes and controls.^23^ In a study by Ermis et al.^24^ a significant decrease in ACD was described when patients with PEX changed from supine to prone position, illustrating the zonular weakness and lens instability in these eyes.

However, in the Reykjavik Eye Study, PEX was not found to be related to CCT, ACD, LT, nuclear lens opacification, or optic disc morphology in a multivariate model.^25^ Doganay et al.^26^ evaluated the anterior segment parameters of patients with PEX syndrome and PEX glaucoma with the Pentacam-Scheimpflug imaging system. Although they found a significant difference in ACD between PEX glaucoma eyes and normal eyes, it was unlikely to be of clinical significance. There were no significant differences in the means of anterior chamber volume, anterior chamber angle width, CCT, PD or in the central 3.0, 5.0 and 7.0 mm corneal volume values among the 3 groups. Oltulu et al.^27^ reported that none of the anterior segment parameters measured by the Pentacam-Scheimpflug imaging system differed between PEX eyes and control eyes. Mean ACD values were 2.7±0.3 mm in the control group, 2.7±0.3 mm in PEX eyes without glaucoma and 2.6±0.4 mm in PEX eyes with glaucoma (p>0.05). In our study, although mean ACD was lower and LT was higher in eyes with PEX syndrome (2.86 mm and 4.60 mm, respectively) and PEX glaucoma (2.90 mm and 4.62 mm, respectively) compared to the control group (2.99 mm and 4.46 mm, respectively), the differences were not statistically significant (p=0.53 and 0.06, respectively). None of our PEX patients had clinically remarkable zonular and lens instability or dense cataract in which OLCR was unable to take measurements. All of the PEX eyes with and without glaucoma had open angles and the axial lengths were similar among the three groups, which might also affect the anterior chamber parameters.

As adequate mydriasis is a main prerequisite for cataract surgery, the PD value is of utmost importance. In PEX syndrome, the PD was found to be smaller and intraocular ciliary muscle function was shown to be disturbed.^1,28^ It takes more time for pupillary dilation in eyes with pupillary-lenticular exfoliation compared to eyes with only pupillary pseudoexfoliation.^29^ In a study by Bosnar et al.,^7^ PD measured in primary position with Lenstar LS 900 was significantly smaller in hypermetropes of the PEX group (3.93±0.84 mm) compared to the hypermetropes of the control group of patients (4.30±0.87 mm) (p=0.03), whereas Doganay et al.26 could not detect any differences between the PEX syndrome, PEX glaucoma and control groups. In our study, mean PD in eyes with PEX glaucoma was smaller (4.83 mm) than the PEX syndrome (5.20 mm) and control groups (5.42 mm); however, the difference was not statistically significant (p=0.15).

In many studies evaluating the corneal endothelial changes in PEX syndrome, ECD was found to be lower than non-PEX eyes.^15,16,17,18,19,30,31^ In a study by Quiroga et al.,^17^ specular microscopy was performed on 61 eyes with PEX syndrome and senile cataract and 356 eyes with only cataract in Paraguay. Adjusting for age, only the mean difference in ECD between the groups with and without PEX was significant (PEX=2,315 cells/mm2, no PEX=2,482 cells/mm2, p=0.002). Of the total study population, at-risk ECD of less than 2,000 cells/mm2 was found in 46 eyes (11%), and in 21.87% of PEX eyes. When PEX was present, the calculated odds ratio for corneal decompensation following surgery was 1.90 after adjustment for age. Inoue et al.^18^ examined the corneal endothelium in 26 PEX eyes (7 eyes of glaucoma patients, and 19 eyes of patients without glaucoma) and compared the results with 30 non-PEX eyes with senile cataract or refractive errors who served as the control group. The corneal ECD was significantly lower in the PEX eyes (2,336±383 cells/mm2) than in the non-PEX eyes (2,632±327 cells/mm2) (p=0.003), whereas no significant differences were found in the rates of polymegathism and pleomorphism. Recently, a confocal study showed that eyes with PEX syndrome had significantly lower cell densities in the basal epithelium (p=0.003), anterior stroma (p=0.007), intermediate stroma (p=0.009), posterior stroma (p=0.012), and endothelium (p<0.001) than in the corresponding layers of normal eyes.^19^ Wang et al.^30^ found decreased ECD (2,298±239 cells/mm2) in PEX eyes compared to cataract eyes (2,652±18 cells/mm2) (p=0.026), with no significant differences in coefficient of variation of cell size and frequency of hexagonality between these two groups. In another study using specular microscopy, the corneal ECD was found to be significantly decreased both in the eyes with PEX (26 eyes) and in the clinically unaffected fellow eyes (17 eyes) compared to the normal control eyes (27 eyes) (p<0.001, p<0.01).^31^ The aqueous flare intensity significantly increased in PEX syndrome (p<0.01), showing an inverse correlation with the corneal ECD.^31^ We also found that PEX glaucoma eyes had lower ECD in control eyes (p=0.02) and 15.8% had ECD less than 2,000 cells/mm2. There were no differences in endothelial cell morphology between eyes with and without PEX syndrome, similar to the findings of Inoue et al.^18^ and Wang et al.^30^ Endothelial cell loss might be due to PEX syndrome, cataract, increased IOP or use of glaucoma medications. As mean ECD was lowest in PEX glaucoma eyes, increased IOP and use of glaucoma medications seem to increase endothelial cell damage in addition to PEX syndrome. As all of our glaucoma patients (19 subjects) were on medical therapy, a comparison could not be made between glaucoma patients with or without treatment. We also could not compare the endothelial cell numbers according to number of anti-glaucoma medications or different anti-glaucoma agents, as the numbers of glaucoma subjects within groups were too small.

In contrast, some studies could not find a decrease in ECD in eyes with PEX syndrome, but showed some morphological changes in shape and size.^20,21,22^ In a study by Wali et al.^21^ pleomorphism and polymegathism measured by Confoscan 2 (Nidek, Japan) were found to be more associated with PEX glaucomas (R(2)=0.7652, p=0.02) than with PEX cataracts (R(2)=0.6041, p=0.06). In another study by Wali et al.^22^ the overall mean ECD of 126 PEX eyes of 69 patients with a mean age of 63.19±7.55 years was 2,465.86±506.68 cells/mm2. The endothelial cell count of PEX eyes in this study was found to be within the normal range at 86.5%. The mean values for pleomorphism and polymegathism were 34.63±11.92% and 58.73±16.61%, respectively, which were both abnormal when compared with cut-off values for being normal. Neither the mean ECD nor polymegathism rate differed between PEX eyes with and without glaucoma. However, they did not compare the parameters of PEX eyes with age-matched eyes without PEX syndrome.

In conclusion, although we found no differences in anterior segment biometric parameters, eyes with both PEX glaucoma and cataract seem to be associated with decreased endothelial cell number, which necessitates specular or confocal microscopy screening before intraocular surgery.

## Figures and Tables

**Table 1:**
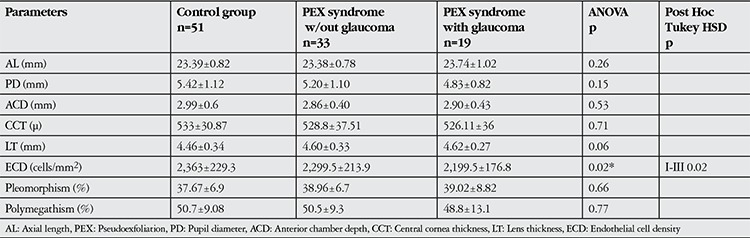
Mean anterior segment parameters and cornea endothelium parameters of eyes with pseudoexfoliation syndrome and senile cataract in comparison with control eyes
